# The Never-Ending Presence of *Phytophthora *Species in Italian Nurseries

**DOI:** 10.3390/pathogens12010015

**Published:** 2022-12-22

**Authors:** Chiara Antonelli, Margherita Biscontri, Dania Tabet, Anna Maria Vettraino

**Affiliations:** Department for Innovation in Biological, Agro-Food and Forest Systems (DIBAF), University of Tuscia, 01100 Viterbo, Italy

**Keywords:** *Phytophthora*, Italy, nursery, Europe, plant trade, biosecurity, biological invasions, plant disease, plant pathogens

## Abstract

Plant trade coupled with climate change has led to the increased spread of well-known and new *Phytophthora* species, a group of fungus-like organisms placed in the Kingdom Chromista. Their presence in plant nurseries is of particular concern because they are responsible for many plant diseases, with high environmental, economic and social impacts. This paper offers a brief overview of the current status of *Phytophthora* species in European plant nurseries. Focus was placed on Italian sites. Despite the increasing awareness of the risk of *Phytophthora* spread and the management strategies applied for controlling it, the complexity of the *Phytophthora* community in the horticulture industry is increasing over time. Since the survey carried out by Jung et al. (2016), new *Phytophthora* taxa and *Phytophthora*-host associations were identified. *Phytophthora* *hydropathica*, *P. crassamura*, *P. pseudocryptogea* and *P. meadii* were reported for the first time in European plant nurseries, while *P. pistaciae*, *P. mediterranea* and *P. heterospora* were isolated from Italian ornamental nurseries. Knowledge of Phytophthora diversity in plant nurseries and the potential damage caused by them will help to contribute to the development of early detection methods and sustainable management strategies to control *Phytophthora* spread in the future.

## 1. Introduction

*Phytophthora* species are Oomycetes, classified within the Stramenopile lineage; they consist of soilborne and airborne species and require water to complete their life cycles. They produce infectious propagules, including zoospores, chlamydospores and oospores, that can be spread short or long distances. These structures enable long-term survival (oospores) and short-term survival (chlamydospores) facilitating the adaptation of *Phytophthora* taxa to different environments [1,2,3,4]. Some *Phytophthora* species are aggressive pathogens that can cause damping-off, root and collar rot, wilting and blight on over 1000 plant species [1]. While some taxa, such as *P. infestans* Mont. de Bary, have a limited range of hosts, species such as *P. ramorum* Werres, de Cock and Man in ’t Veld, can infect more than 109 plant species [5,6,7,8,9,10]. There is a longstanding concern about the presence of *Phytophthora* infestation in plant nurseries. The occurrence of *Phytophthora* spp. is a challenge not only because of the direct economic losses caused to the horticulture industry but also because it poses a threat to biodiversity of woodlands and natural or urban ecosystems, where it can be introduced through reforestation and restoration programs. These pathogens invade new territories through plant trade, as also happens for many other plant pests [11,12,13,14]. *Phytophthora ramorum*, for example, which is responsible for the death of millions of tanoaks (*Notholithocarpus densiflorus* Hook. and Arn.) and *Quercus* spp. in the USA and larch (*Larix kaempferi* Lamb.) in plantations in the UK, spread throughout North America and Europe via the nursery trade [15,16,17,18,19,20]. In Europe, symptoms of foliar blight and dieback were first described on *Rhododendron* and *Viburnum* potted plants in Germany (1995) and the Netherlands (1993) [21]. It was only in 2001 that the causal agent of these diseases and symptoms, associated with the Sudden Oak Death in North America, was described as *P. ramorum* [21]. The number of host plants threatened by *P. ramorum* currently amounts to some 120–130 species, but their list is continuously being revised and updated. The last record in chronological order was from 2021 [22]. Another notable *Phytophthora* species is *P. lateralis* Tucker and Milbrath, which was isolated from nurseries in Washington State before spreading through forests of *Chamaecyparis lawsoniana* A. Murray bis Parl in Oregon [23]. Similarly, *P. tentaculata* Kröber and Marwitz, spread from native plant nurseries onto restoration sites [24,25,26].

In plant nurseries, *Phytophthora* species have optimal environmental conditions for their growth and spread due to the presence of many different plant species in suitable temperature and humidity conditions within a favorable survival range. Poor hygienic practices, e.g., reusing non-sanitized containers, splashing and *Phytophthora* contaminated irrigation water, in most nurseries offer many opportunities for the spread of the pathogen within the production areas [11,27]. In addition, the most common fungicides applied in plant nurseries, mefenoxem and fosetyl-Al, have a fungistatic effect, thus plants can appear healthy but already be infected. Therefore, ordinary visual inspections can fail to detect the pathogen [12,28,29,30]. At the same time, the inappropriate use of chemicals can result in the development of resistant strains [31,32]. In plant nurseries, interspecific hybridization events can also occur. The co-occurrence of taxa, previously geographically isolated, in the same environment/host can favor the development of hybrids, which can be more virulent than their progenitors. Relevant examples are *P. alni* Brasier and Kirk, a hybrid between *P. cambivora* Petri Buisman and a *P. fragariae*-like species, which, alone in 1996, killed over 10,000 riparian *Alnus* trees in Europe [33]. Similarly, *Phytophthora* × *pelgrandis* Gerlach, Nirenberg and Gräfenhan, a hybrid between *P. nicotianae* Breda de Haan and *P. cactorum* Lebert and Cohn J. Schr., infected several plants [34].

The impacts of *Phytophthora* introduction may be multiple due to their ability to colonize forests, ornamental plantings and nurseries and to infect different plant hosts [35,36,37,38,39,40,41,42]. *Phytophthora nicotianae*, just to cite a single example, one of the most common *Phytophthora* taxa in plant nurseries, alone infects over 255 genera in 90 plant families and is present in European nurseries, forests and landscape plantings [35,43]. Once a pathogen is established in an environment, its eradication requires huge efforts and costs that can easily exceed USD 100 billion per year [44]. In addition, history has shown that introductions of Phytophthoras have also caused political and social unrest, and even famine, pestilence and death, as happened with the potato late blight caused by *P. infestans*. The Spanish introduced potatoes into Europe after their conquest of the New World. First introduced in Belgium in 1844 [45], *P. infestans* rapidly spread to other European countries. The consequent devastating epidemic killed about 700,000 people and forced a similar number of people to leave Ireland, where the subsistence of the population was mainly based on potatoes [46,47]. Late blight remains the most destructive disease of this plant species. 

Clearly, the prevention of the introduction of pathogens by using healthy plants is the most effective approach to deal with threats caused by *Phytophthora* species. In this context, it is crucial to have a clear picture of *Phytophthora* species diversity currently present in plant nurseries.

## 2. *Phytophthora* Species Diversity in Plant Nurseries

Numerous *Phytophthora* species have been documented in commercial plant nurseries worldwide, causing significant economic losses [1,48,49,50,51]. For instance, a total of 28 and 15 *Phytophthora* taxa, have been found in Oregon and California ornamental nurseries, respectively [52,53]. Some of the *Phytophthora* taxa found in plant nurseries are of regulatory concern. Among those, *P. ramorum* has received the most notoriety, but also other regulated taxa can be present. The first European detection of *P. lateralis* (EPPO List) is dated 1999 in French nurseries [54]. Of over 36 *Phytophthora* species identified in Pennsylvania nurseries and greenhouses, three, *P. parvispora* Scanu and Denman; *P. chrysanthemi* Naher, Watanabe, Chikuo and Kageyama; and *P. sojae* Kaufm. and Gerd., were listed in the U.S.-regulated Plant Pest Risk [55,56]. During 1972–2013, an intensive survey was conducted of 732 nurseries and over 2525 forest and landscape plantings in 23 European countries [35]. A total of 65 *Phytophthora* taxa, from nine clades, were recorded in association with more than 600 hosts. *Phytophthora plurivora* Jung and Burgess, *P. cinnamomi* Rands, *P. nicotianae*, *P. cryptogea* Pethybr. and Laff. and *P. cactorum*, were the most isolated in forest and landscape plantings, while *P. plurivora*, *P. cinnamomi*, *P. cactorum*, *P. nicotianae*, *P. ramorum* and *P. citrophthora* R.E. Smith and E.H. Smith Leonian were observed from most of the plant nurseries surveyed. Several hosts could be associated with different *Phytophthora* species. *Rhododendron* alone was threatened by 12 different *Phytophthora* taxa in ornamental plantings in Croatia, Germany, Hungary, Italy, Spain, Sweden, Switzerland and the United Kingdom. Since Jung et al. [35], European researchers published additional reports of newly recorded *Phytophthora* taxa and new *Phytophthora*-host associations. For the first time, *P. crassamura* Scanu, Deidda and Jung was detected in Spain from potted *P. pinea* L. seedlings [57] Nonetheless, the pathogenicity of *P. crassamura* on *P. pinea* was not proven. Authors also associated *P. pseudocryptogea* Safaiefarahani, Mostowfizadeh, Hardy and Burgess, with the new hosts *Chamaecyparis lawsoniana* and *Yucca rostrata* [58]. 

Some *Phytophthora* taxa observed in European nurseries are not yet identified at the species level, such as *P. cryptogea*-like strains [59] and *Phytophthora* sp. 1, closely related to *Phytophthora meadii* McRae and *P. citrophthora*, obtained from Spanish plant nurseries [57,59].

### 2.1. The Case of Phytophthora Species in Italian Plant Nurseries

The survey conducted by Jung et al. [35] described a total of 36 different *Phytophthora* taxa in Italian plant nurseries, forest and landscape plantings. Those taxa were not exclusive to Italy, confirming nurseries as a potential basin of plant pathogens [35,60]. 

*Phytophthora cinnamomi*, *P. cambivora* and *P. cryptogea* occurred mainly in oak stands, while *P. palmivora* E.J. Butler was isolated from all nurseries stands of *Olea europaea* L. Over more than 100 *Phytophthora*-host associations, 33 new hosts were reported exclusively in Italy, including *Agave attenuata* Salm-Dyck., *Coronilla valentina* L. and *Solanum melongena* L. *Phytophthora ramorum*, *P. fragrariae* Hickman and *P. lateralis* were the only three *Phytophthora* species recorded in Italian plant nurseries and plantings also included on the EPPO quarantine list. Interestingly, *P. ramorum* was first isolated in Italy in 2002, on *R. yakushimanum* Ken Janeck in a Piedmont nursery [61]. Later, in 2013 the pathogen was detected by pyrosequencing analysis in chestnut stands and by culture-based methods on *Viburnum tinus* L. in Pistoia, where currently it is considered eradicated [62,63]. Nevertheless, its record in Italian plant nurseries confirmed that it was not halted despite strict quarantine regulations. 

In this study, a literature survey was carried out using databases available for academic research, such as Scopus [64] and Web of Science [65], using “*Phytophthora* and Italy” as keywords. The dataset was then restricted to nurseries. Results highlighted the presence of 43 *Phytophthora* species associated with horticultural, forest and ornamental plant species. The richness of *Phytophthora* taxa in Italy could be associated with the geographic characteristics of the country and its extensive trading traditions. 

**Figure 1:**
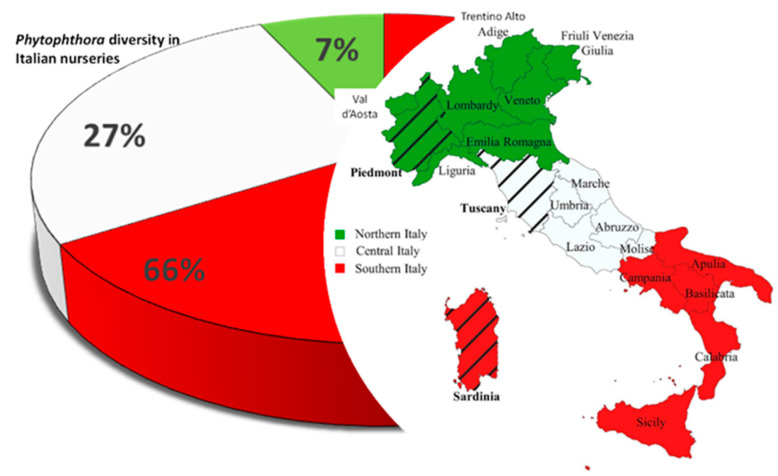
Distribution map of *Phytophthora* spp. in Italian plant nurseries. Different colors represent the three Italian zones: red = Southern Italy; white = Central Italy; green = Northern Italy. The regions within each zone with the highest *Phytophthora* diversity are indicated by stripes.

Italy is a long peninsula with mostly mountainous hinterland and is surrounded on every side except the north by the Mediterranean Sea. Thus, its climate is highly diverse. The lack of information of the exact geographic locations of the nurseries reported in the dataset analyzed has prevented us from highlighting a possible link between *Phytophthora* diversity and climate conditions. Nevertheless, the diversity of the *Phytophthora* community decreased from Southern to Northern Italy (Figure 1). Sardinia, Tuscany and Piedmont were the regions with the highest *Phytophthora* spp. richness in the three Italian zones (Southern, Central and Northern Italy, respectively) (Figure 1). *Phytophthora ramorum*, *P. cinnamomi*, *P. nicotianae* and *P. niederhauserii* Abad were present throughout the peninsula. However, the structures of *Phytophthora* communities varied in the three zones also in accordance with the distribution of hosts and efforts to characterize occurrence, with most of the survey conducted after 2000. The rising outbreaks of *Phytophthora* species in forests and natural ecosystems in Europe in the late 1990s probably stimulated the scientific community to investigate with systematic surveys the presence of pathogens in nurseries.

**Figure 2:**
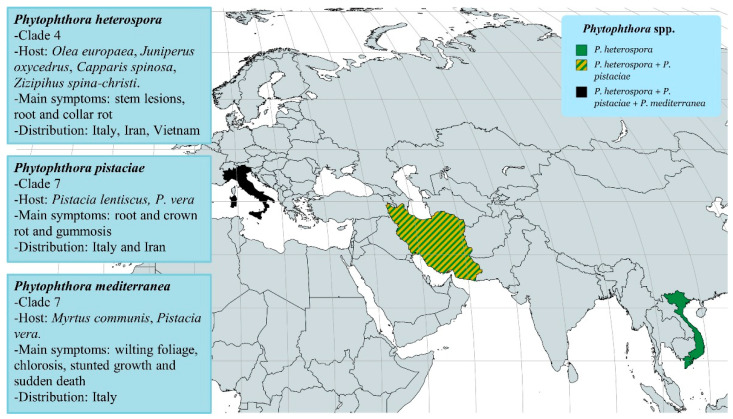
Worldwide geographical distribution of *P. pistaciae*, *P. mediterranea* and *P. heterospora*.

The previous survey by Jung et al. [35] focused on data collected from 1992 to 2013. Since then, three new *Phytophthora* species, *P. pistaciae* Mirabolfathy; *P. mediterranea* Bregant, Mulas and Linaldeddu; and *P. heterospora* Scanu, Cacciola, Linald. and T. Jung, were described in Italy. These were the first reports in Europe (Figure 2). 

*Phytophthora mediterranea* was isolated from declining potted myrtle seedlings (*Myrtus communis* L.) in Italy [66]. It was previously observed on pistachio (*Pistacia vera* L.) in California [67]. Although phylogenetically, *P. mediterranea* is closely related to *P. cinnamomi*, the two species can be easily distinguished on the basis of some morphological differences, such as size of the sporangia, colony growth pattern and cardinal temperature values. Several Mediterranean maquis species are highly susceptible to this newly recognized pathogen [66].

*Phytophthora pistaciae* causes leaf reddening, wilted shoots, root and collar rot on nursery plants of *P. lentiscus* L. in Italy [68]. It is considered the most aggressive pathogen of *P. vera* in Iran [69,70].

*Phytophthora heterospora* has been isolated from stem lesions and root and collar rot of *Olea europaea* (2010, Italy), *Ziziphus spina-christi* L. Desf (2011, Fars Province, Iran), *Juniperus oxycedrus* L., *Capparis spinosa* L. (2013–2014, Italy) and *Durio zibethinus* L. (2013, Mekong River delta, Vietnam) [71]. *Phytophthora heterospora* and *P. palmivora* have many similar morphological characteristics in terms of colony morphology, sporangia, chlamydospores, and gametangia shape and size. However, *Phytophthora heterospora* produces pseudoconidia, a unique asexual dissemination structure of *Phytophthora* species. This feature was previously described on isolates obtained from *Theobroma cacao* L. in the Ivory Coast and named *P. palmivora* var. *heterocystica* Babacauh [72]. Unfortunately, it is unclear whether P. heterospora and *P. palmivora* var. *heterocystica* represent the same taxon. 

Pathways of *P. heterospora* and *P. pistaceae* introduction are unknown. It is worth noting that Italy has a long history of trade in goods with Iran, where both *P. heterospora* and *P. pistaceae* are present. It is the second-largest importer of shelled pistachios in Europe (after Germany), with a value of USD 193 million in 2020 [73]. 

Considering the high virulence of *P. mediterranea*, *P. pistaciae* and *P. heterospora*, their discovery could seriously damage Mediterranean crops. Just to focus on a few examples, we could easily imagine the consequences of their spread into myrtle and olive tree areas. Myrtle is native to Europe but is widespread throughout the Mediterranean and the Middle East regions. It is widely cultivated as an ornamental plant, used in medicine, and to produce an aromatic liqueur called “Mirto” in Sardinia (Italy) and Corsica (France); it is also seen as a symbol of restoration and recovery. The olive (*Olea europaea*) is a well-known evergreen tree native to the Mediterranean coast. Global statistics indicate that olive is cultivated on an area of approximately 10 million hectares, with over 90% located in the Mediterranean Basin. Olive fruits and oil are used for food and cooking. In 2021/22 alone, the global utilization volume of olive oil amounted to just under 3.2 million metric tons [73]. Thus, it is evident that spread of the previously cited new *Phytophthora* taxa through olive and myrtle plantations will have detrimental effects on landscapes leading to social and economic consequences.

Since 2013, the list of *Phytophthora*-host combinations was reviewed with novel associations including *P. psychrophila* Jung and Hansen/*Ilex aquifolium*, *P. pseudosyringae*/*I. aquifolium*, *P. pseudocryptogea*/*Laurus nobilis* L., *P. megasperma Dreschsler*/*L. nobilis*, *P. citrophthora*/*L. nobilis*, *P. bilorbang* Aghighi and Burgess/*Phyllirea latifolia* and L. and *P. palmivora*/*P. latifolia* [66]. *Phytophthora* × *pelgrandis*, previously observed in potted plants in the Netherlands, Hungary and Germany, and *Phytophthora hydropathica* Hong and Gallegly, previously never detected in European nurseries, were also reported on *Lavandula* spp., *Buxus sempervirens* L., *C. lawsoniana* and *Viburnum tinus* L. in Italy [74,75,76]. During 2012–2014, *P. pseudosyringae* was first detected using ITS DNA metabarcoding [77,78] and only in 2021, was isolated from potted plants of *Ilex aquifolium* L. in Sardinia (Italy) [66]. In all probability, this is only the beginning of the story; the final picture of the occurrence of *Phytophthora* species in plant nurseries will probably never be complete, as additional species are being discovered every year. Researchers believe that the diversity of *Phytophthora* species presently is well underestimated. Although a total number of 200 *Phytophthora* taxa have been described [71,79], another 200–400 species may remain to be discovered in environments not yet surveyed [80] or not yet formally identified [77,78]. 

### 2.2. Detection Methods for Phytophthora Species in Italian Nurseries 

The most applied technique to describe the *Phytophthora* community in European nurseries was a culture-based detection system, often preceded by baiting of soil and root samples [35,66,68,71]. This method is based on the ability of motile zoospores released from sporangia to swim upwards and towards living baits. Once infected, baits can show symptoms, often consisting of black spots or small lesions. Fragments of infected tissues are then plated on synthetic media [1]. Fungal-like isolates are then analyzed on the basis of morphological and molecular characteristics. 

Interestingly, the type of baits chosen is linked also to their practical availability. In the studies related to the *Phytophthora* occurrence in European nurseries, while leaves of *Quercus* spp. and *Rhododendron* spp. were used as baits in almost all isolation protocols applied, *Citrus* spp. and *Sambucus nigra* leaf baits were reported only for southern Europe [35,66,68,71]. *Phytophthora* isolates were obtained also by both direct isolation from stem lesions, infected roots, and collar tissues and plate dilution methods [35,66] using BNPRAH (Potato dextrose agar 1%, 20 mg benomyl, 25 mg nystatin, 25 mg PCNB, 10 mg rifampicin, 500 mg ampicillin, 25 mg hymexazol, in 1 L of deionized water), PARPNH (V8-juice 200 mL, 10 ppm pimaricin, 200 ppm ampicillin, 10 ppm rifampicin, 25 ppm quintozene, 50 ppm nystatin, 50 ppm hymexazol, Potato dextrose agar 15 g agar, and 3 g CaCO_3_, in 1 L of deionized water), SMA (40 g dextrose, 2 g asparagine, 0.5 g KH_2_PO_4_, 0.25 g MgSO 7H_2_O, 0.5 g thiamine chloride, and 15 g agar, in 1 L of deionized water) and PDA+ (Potato dextrose agar 39 g, 100 mL of carrot juice, 0.013 g pimaricin, and 0.05 g hymexazol, in 1 L of deionized water) media [35,68].The culture-based approach is a robust methodology for *Phytophthora* isolation but may underestimate the pathogen community diversity due to sampling bias, type of baits or isolation media used, and the seasonality and types of propagules in the sample [1,37,81]. For instance, a selective medium can inhibit the taxa more sensitive to specific compounds [82]. Burgess et al. [83], applying the same isolation protocols on different substrates, showed that *Phytophthora* isolation efficacy can be affected by the types of soil studied and the complex of *Phytophthora* species present. Moreover, the identification of *Phytophthora* species is difficult since some taxa show similar morphological characteristics that may not be distinguishable to those with insufficient experience. Due to the different variables involved, it is difficult to provide a standard protocol for *Phytophthora* isolation. However, some authors identified troubleshooting steps that may be included carefully to enhance *Phytophthora* detection [83,84]. Lastly, the culture-based method requires basic knowledge of cultural techniques and involves long incubation and growth times. 

Molecular techniques based on sequencing of specific regions of DNA are required to complement morphological characterizations. Historically, sequencing of the ITS region was used to detect *Phytophthora* in European nurseries [35]. However, due to ITS sequence homology in some *Phytophthora* species, for example *P. rubi* and *P. fragariae*, a multigene phylogenetic analysis of the genus is more commonly conducted [66,68]. The resolution of different markers can vary with species and sub-clade. Thus, a two-step approach is recommended: using ITS as the first marker followed by identification to species level using one or more of the most informative markers for the respective (sub)clade [85]. Over the last 15 years, there has been intensive research into the development of rapid, specific and sensitive detection tools for *Phytophthora* species [86,87,88,89,90]. Generally, the protocols were geared to one or few specific pathogen species, a major weakness when used in plant health surveys. Conversely, recently developed tests offer the advantages of being specific to the genus *Phytophthora* or applicable entirely in the field, independent of any laboratory facilities, providing the rapid answers required by the market [91,92,93]. On-site methods, also called point-of-care (POC), provide a preliminary screening but do not replace laboratory testing, which remains crucial for more complex research, such as identification and classification of new pathogens or the study of plant defense mechanisms. Due to the significance of a finding of *Phytophthora* species, a clear understanding of performance characteristics of diagnostic assays is crucial, and guidelines should be developed for accurate pathogen detection from different substrates. Recently, high throughput sequencing (HTS) technology has been proposed as a phytosanitary assessment tool for the detection of *Phytophthora* in Italian nurseries. Metabarcoding allows an accurate description of the microbial community, detecting rare *Phytophthora* species, the isolation of which in culture may be hindered by faster growing or more abundant species. In accordance with studies conducted using isolation-based methods, metabarcoding showed a very complex assemblage of *Phytophthora* taxa and the common occurrence of P. nicotianae in ornamental nurseries [77,78]. The HTS approach revealed potential new taxa, such as *P. meadii*-like, *P. cinnamomi*-like and *P. niederhauserii*-like, the presence of which needs to be confirmed by isolation methods [77,78]. Unfortunately, discrimination between *Phytophthora* species using HTS, generally based on ITS region analysis, can be challenging due to the low genetic variation and the absence of reliable databases [86]. For instance, Prigigallo et al. [77,78] attributed three phylotypes to two or more taxa (*P. citricola* taxon E or III; *P. pseudosyringae*, *P. ilicis* Buddenhagen and Young or P. nemorosa Hansen and Reeser and *P. cryptogea*, *P. erythroseptica* Pethybr., *P. himalayensis* Dastur or *P. sp.* ‘*kelmania*’), a riddle that cannot be solved without examination of pure cultures. Comparative studies on *Phytophthora* population compositions obtained by culture-dependent and metabarcoding methods showed that both techniques complemented each other [5,94,95,96]. Currently, several web-accessible databases have been developed to support accurate and rapid identification of *Phytophthora* species (http//www.phytophthora-id.org, http//www.phytopathdb.org and http//www.boldsystems.org, accessed on 19 December 2022). The systematic cataloging of genotypic and phenotypic information of previously described species, the sequencing of several genomes (http://www.ncgr.org, accessed on 19 December 2022) and the practical guides for *Phytophthora* detection and identification provide a solid basis for diseases monitoring. Continuous updating of these resources and an improvement of identification and diagnostic protocols promise a more detailed understanding of *Phytophthora* distribution in the near future.

## 3. How to Tackle the Spread of *Phytophthora* Species

Before the COVID-19 pandemic, the value of horticulture production in Italy exceeded EUR 2.7 billion. Regarding plant nurseries, the figures also included cut flowers and flowering plants. During the periods of lockdown, all seasonal products were irremediably lost due to the impossibility of watering, for a short period, and the demand for ceremonies and anniversaries. Fortunately, matters are gradually improving for this sector in both the domestic and foreign markets. The return to gardening practices has led to an appreciable increase in sales. Export, however, is the driving force for the sector, with a value of about USD 28,765,318.00 [97], with demand coming mainly from Northern European countries (Holland, Germany and France). Italian imports of plants and live plant materials (import values USD 86,437,699.00; data 2020) comes mainly from The Netherlands (71%), Germany, Spain and Poland [97]. In globalized trade, plants and plant products are continuously on the move. Marketing has switched from conventional to web-commerce sites, exacerbating potential phytosanitary risks as delivery often bypasses traditional screening by NPPOs [98,99,100].

Not least, the distribution of pests is clearly altered by climate change. The presence of *P. cinnamomi* in alpine areas is emblematic. Its quick spreading in new geographic areas was reported in forests [41,101,102] as well as in German nurseries, where generally it is rare due to its sensitivity to frost [103]. In this scenario, the future of *Phytophthora* spp. occurrence is dangerously uncertain. 

Addressing the risks of *Phytophthora* spread is a highly complex task. Despite good intentions to control pest introduction and spread, we must be aware of the weakness and the lack of harmonization of phytosanitary regulations and processes [14,28,100,104,105,106,107,108]. The recently adopted new Plant Health Regulation (EU) 2016/2031, enhancing more effective measures for the protection of the Union territory and its plants, ensures safer trade, as well as proposing mitigation measures for the impacts of climate change on the health of crops and forests. The application of the new law cannot tackle the issue alone. It is essential to develop pest risk assessments that underpin policy and decision-making to assess the risks of introduction, spread and the environmental impact posed by invasive alien species (IAS). However, during the introduction steps, pathogens could be particularly hard to identify. They can express a pathogenic lifestyle only following introduction into new areas and in association with new hosts. Several guidelines and protocols for risk assessments have already been drawn up, but an effort to harmonize them and enhance communication and information exchanges with other countries is suggested [109,110,111].

The development of new rapid, reliable, accurate and cost-effective detection methods is also widely desirable to prevent spread of *Phytophthora* spp. Apart from molecular approaches, such as environmental DNA metabarcoding, aerobiology or the use of sentinel plants, represent a challenging but helpful research line for bio-surveillance of IAS [112,113,114]. 

Once in a nursery, spread of *Phytophthora* is difficult to stop. Several guidelines were published to help to maintain a nursery system that excludes *Phytophthora* pathogens [115,116]. The application of those protocols, however, could be hampered by practical issues. They could require technical practices, such as testing irrigation water for the presence of pathogens, which represent additional costs for professional growers. In this context, it is important to inform professionals in the sector of the risk and consequences of plant diseases that are often hidden by chemical treatments. 

It is increasingly recognized that surveillance activities should be developed for early detection both in the areas of interest and in the exporting regions outside the EU. Thus, field workers and inspectors at borders should continuously update their knowledge or skills to recognize symptoms of plant diseases. In recent years, several molecular methods have been developed for early detection of *Phytophthora*; however, they often require expertise not generally present in plant nurseries, meaning that growers need to pay for external services. It is a matter of fact that plant nurseries are generally small-sized enterprises, about 1.3 ha/nursery in Italy, that could hardly bear the costs of biosecurity strategies, despite the necessity. External financial aid, for example from EU plant health organizations, could support bio-surveillance practices.

Among the strategies suggested for *Phytophthora* disease management, the biological protection approach results in one of the most eco-sustainable control methods by inhibiting plant pathogens, improving plant immunity and/or stimulating microorganisms beneficial to the plants. Gaining a better understanding of the interaction of biological control agents with the environment and the development of new eco-friendly products, such as nanoparticles as carriers of plant extracts or other chemicals [117,118,119,120], will be important for the improvement of environmentally sustainable management protocols. 

Given the global nature of *Phytophthora* disease problems, bio-surveillance should be introduced encompassing global cooperation in monitoring, detection, studying and managing the pathogen. Encouragement of better collaborations among research centers, growers and national and international organizations will optimize efforts for protecting plants. Moreover, a reciprocal exchange dialogue is required with the public and industry to work in synergy in order to fully share common control strategies, increase awareness of the risks in plant trade and the importance of protecting and maintaining local biodiversity. 

## 4. Conclusions

The plant nursery industry is a reservoir for *Phytophthora* species, whose spread will be exacerbated by the effects of the ever-increasing global plant trade, climate change, the introduction of highly susceptible or asymptomatic hosts and the emergence of new threats, or a combination of these issues. These factors will have a decisive influence on the geographic distribution of pathogens, their virulence and host range into the future. It is, therefore, not surprising that in the future, new combinations of host-pathogens or new *Phytophthora* hybrids will occur.

The growing number of publications and citations for *Phytophthora* species could be interpreted as an increasing awareness of their environmental, economic and social impacts. Nonetheless, there remains a lack of information about the occurrence of *Phytophthora* spp. in nurseries, illustrating the need to develop simple, efficient early detection methods and management strategies. More efforts should be addressed to highlight the risk posed by new introductions of *Phytophthora* species as a matter of urgency by government agencies, international health organizations, managers, plant nurseries and citizens.

In this scenario, nurseries will play a crucial role. By enforcing appropriate biosecurity practices and early detection, they can reduce their economic losses and limit pest spread into forests and urban areas. This study highlighting the rapid increase in the number of *Phytophthora* species in European plant nurseries will contribute to raised awareness of managers and scientists on the importance of implementing appropriate biosecurity measures to minimize the ecological and economic threat posed to the forest and food chains as well as natural ecosystems and urban areas. 

## Data Availability

Not applicable.
